# A Case Report of *Mycoplasma hominis* Subdural Empyema Following Decompressive Craniotomy, and a Review of Central Nervous System *Mycoplasma hominis* Infections

**DOI:** 10.3389/fmed.2022.792323

**Published:** 2022-02-24

**Authors:** Assaf Potruch, Guy Rosenthal, Ayelet Michael-Gayego, Violeta Temper, Mohanad Abdelrahman, Oshrat Ayalon, Ran Nir-Paz, Yonatan Oster

**Affiliations:** ^1^Department of Internal Medicine, Hadassah Hebrew University Medical Center, Jerusalem, Israel; ^2^Faculty of Medicine, Hebrew University of Jerusalem, Jerusalem, Israel; ^3^Department of Neurosurgery, Hadassah Hebrew University Medical Center, Jerusalem, Israel; ^4^Department of Clinical Microbiology and Infectious Diseases, Hadassah Hebrew University Medical Center, Jerusalem, Israel

**Keywords:** *Mycoplasma hominis*, CNS infection, subdural empyema, multi-trauma, MALDI TOF

## Abstract

**Background:**

*Mycoplasma hominis* is a small cell-wall-free organism, part of the normal microbiota of the genitourinary tract. It is rarely involved in extragenital infections, mainly joint, surgical-site, and respiratory infections.

**Methods:**

We describe a case of *M. hominis* subdural empyema and lower limb surgical site infections, following decompressive craniotomy, after traumatic brain and extremities injury. In addition, a literature review of 34 cases *M. hominis* CNS infections was done.

**Results:**

Our case depicts a 25-years old patient who developed subdural empyema and surgical site infections in his cranium and fibula. Both sites were cultured, and small pinpoint colonies grew on blood agar. MALDI-TOF MS identified *M. hominis*. Simultaneously 16S-rDNA PCR from CSF detected *M. hominis*. Antimicrobial treatment was switched to doxycycline with improvement. Literature review revealed 21 adults and 13 pediatric cases of *M. hominis* CNS infection. Risk factors in adults were head trauma, neurosurgery, or post-partum period.

**Conclusions:**

Based upon the literature reviewed, we postulate that adult patients with head trauma or neurosurgical procedure, rarely are infected either through direct contamination during the trauma, or by undergoing urgent, urinary catheterization, and may experience distant infection due to translocation of *M. hominis* into the bloodstream. In such cases diagnosis is delayed due to difficulties in growing and identifying the bacteria. Empiric antimicrobials are usually not effective against mycoplasmas. These factors contributed to the mortality in adult cases (15%). Our rare case highlights the necessity of combining classical microbiology routines with advanced molecular techniques to establish a diagnosis in complicated cases.

## Introduction

*Mycoplasma hominis*, originally identified in 1937 by Dienes and Edsall (1), is considered part of the normal microbiota of the genitourinary (GU) tract (2). It is a small cell-wall-free intra and extracellular organism.

Infections caused by *M. hominis* are usually limited to the GU tract (3). The bacterium has a symbiotic relationship with *Trichomonas vaginalis* and can live intracellularly in the host and the parasite (4) while receiving its essential metabolites, fatty acids, amino acids, and nucleic acid precursors, from its host. The limited capacity for biosynthesis and lack of cell wall explains their saprophytic lifestyle, their sensitivity to environmental factors and inherent resistance to β-lactams.

*Mycoplasma hominis* was isolated in the vaginal swabs of about two thirds of female patients with vaginal discharge, and only in about 10% of women with normal findings and is strongly associated with bacterial vaginosis (5). It has also been isolated from blood of 13% of women with postpartum fever (6), as well as post-abortion fever (7). In men however, numerous studies done have shown no difference in the prevalence of the organism in urethral specimens of asymptomatic and symptomatic individuals (8).

Typically, *M. hominis* cannot infiltrate beyond the submucosa of the GU tract, however extragenital infections have been described in the past, including post-surgical infections, mainly after cardiac surgery—superficial and deep surgical wound infections, mediastinitis, and pericarditis (9). Other uncommon infections are joint infections, neonatal conjunctivitis, and respiratory tract infections (10).

From the analytic aspect of the clinical microbiology laboratory, since this organism lacks cell wall, *M. hominis* is not identifiable by gram staining. Culture of this fastidious organism requires time and resources—Optimal *in vitro* growth of *M. hominis* requires specialized liquid and solid media containing amino acids, nucleic acid precursors and arginine as a source of energy (11, 12); therefore, many clinical laboratories generally do not use culture methods for the diagnosis of genital mycoplasmas. Among the *Mycoplasma* and *Ureaplasma* species, only *M. hominis* is sometimes detectable using routinely used laboratory methods. *M. hominis* grows slowly on blood agar, producing tiny translucent non-hemolytic colonies after two to seven days of incubation (13). Molecular method such as 16S rDNA PCR amplification is a possible technique for identification of *M. hominis*, however sensitivity is moderate when performed directly from tissue (14–16). In recent years, Matrix-assisted Laser Desorption\ionization Time-of-Flight Mass Spectrometry (MALDI TOF MS) has emerged as a new technology for microbial species identification. It is a rapid, accurate, and inexpensive method for identifying gram-negative and gram-positive bacteria, yeasts and some molds (17); MALDI TOF MS has become a gold standard for microbiological identification in clinical laboratories (18). Pereyre et al. (19) studied the use of MALDI TOF MS for *Mycoplasma* identification and subtyping, and showed that this technology is rapid, reliable, and cost-effective for this purpose. A one-milliliter culture has been shown to be sufficient in identifying *M. hominis* in clinical isolates. Pailhories et al. (20) described a case in which MALDI TOF was used for the diagnosis of *M. hominis* infection of a subdural hematoma. A limitation in the routine clinical microbiology laboratory is that the procedure is usually performed on colonies grown on solid media.

## Case Report

A 25-year-old, previously healthy male, had an electric scooter accident while on vacation in Cyprus, with severe injuries to the extremities and head, including epidural and subdural hematomas in the left fronto-temporal region, with temporal sphenoidal and maxillary fractures. The patient presented to another hospital where he underwent an urgent decompressive craniectomy. An intracranial pressure monitor was placed, and the left tibia and fibula were splinted. A few days after surgical intervention and stabilization, the patient was transferred by air ambulance to our hospital in Israel for further treatment. After ICU admission, he underwent internal fixation of the left tibia and fibula. On the same day, antibiotic treatment with piperacillin-tazobactam was initiated due to ventilator-associated pneumonia. Chest CT showed an infiltrate in right upper lobe. The white blood count (WBC) was 12.7 × 10^9^/L and the C-reactive protein (CRP) was 21.95 mg/DL (0–0.5). Seven days after his admission, a decline in his mental condition was observed, the patient developed a fever of 38.2^C^, yet was hemodynamically stable. In addition, a purulent discharge was seen from the craniectomy wound, and inflammatory markers were elevated (WBC of 13.5 × 10^9^/L, CRP rose to 29 mg/DL on day 8). A lumbar puncture was performed, and cerebrospinal fluid (CSF) had glucose levels of 0.4 mmol/L (normal range 2.5–3.6 mmol/L) and protein of 5,038 mg/L (normal range 80–320 mg/L), cell count showed 30 polymorphonuclear cells, and no lymphocytes or red blood cells were present. Gram stain was negative for any organisms, and empiric treatment with meropenem and vancomycin was started for post-surgical meningitis. According to the laboratory protocol for CSF processing, 10 μl of sample was plated on each of the following: non-selective (chocolate and blood, Novamed, Israel) and selective (MacConkey, Novamed, Israel) agar plates, as well as in liquid enrichment broth (Thio and TSB, Novamed, Israel). Plates were incubated at 35°C with 5% CO_2_ for 5 days, and liquid media were incubated at 35°C on ambient air for 14 days. After 48 h of incubation, small pinpoint colonies appeared on blood agar, and the MALDI TOF (MS VITEK, bioMerieux) identified the bacteria as *M. hominis*. Simultaneously 16S-rDNA PCR from CSF detected *M. hominis*. Antibiotic treatment was switched to doxycycline. On the same day, the patient developed surgical site infection of both his left cranium and his left fibula; both sites were drained, and swabs were sent to the laboratory for cultures. Similar pinpoint colonies appeared in these as well, and a diagnosis of *M. hominis* infection was made in both surgical sites. Interestingly, blood cultures taken during this event were strile.

Doxycycline treatment continued for 28 days, and slow neurological improvement was seen with a difficult and prolonged post-operative course. The patient's inflammatory markers improved. CRP after 14 days of treatment was 0.4 mg/DL, with a similar response of WBC, which reduced to 5 × 10^9^/L. Later the patient underwent cranioplasty, discharged to rehabilitation facility and in last follow-up was speaking and walking with assistance.

Data collection was done under an approved protocol of the Hadassah Medical Center Ethics Committee (HMO-460-12), including a waiver of informed consent.

## Literature Review

*Mycoplasma hominis* has been described in the past as a pathogenic organism implicated in CNS infection, either meningitis or brain abscesses (21). A combined PubMed search for “mycoplasma hominis,” and “CNS infection” or “encephalitis” or “Meningitis,” without restriction of publication date, was performed to assess the number of cases previously described and yielded a combined total of 76 results. After filtering in all case reports and eliminating duplicates, 33 articles were left describing 34 cases of central nervous involvement of *M. hominis*.

Using the Joanna Briggs Institute (JBI) Critical Appraisal Checklist for Case Reports (22), we assessed the quality of case reports included in this study by seven checklist points, with the point about adverse/unanticipated events reduced. All adult cases included in this study checked at least 5/7 points on the checklist with most of them checking all the checklist points.

The first case description was in 1950 by Paine et al. (23), since than, 21 cases describing adult patients (Table 1) and 13 pediatric patients (Table 2). Among the 21 adults, ages varied from 17 to 76, with a mean age of 33. Half of the cases were females. There is no documented mechanism for the occurrence of this organism in a specific individual. All affected adults had contributing factors such as head trauma, neurosurgical intervention, and the postpartum period in women. One of the main theories is that patients that underwent head trauma or neurosurgical intervention, had also undergone urgent urinary catheterization or oro-tracheal intubation, which can cause seeding of *M. hominis* to the bloodstream either from the genitourinary tract or from manipulation of the respiratory tract mucosa, potentially resulting in infection (30). Immunodeficiency, whether cell- or antibody-mediated, has been linked with excess risk for extragenital infection with *M. hominis* (10).

**Table 1:** Adult cases of *M. hominis* CNS infection.

**Case**	**Age**	**Sex**	**Sample site**	**Type of infection**	**Pre-disposing factor**	**Microbiology diagnosis**	**References**
1	20	M	CSF + abscess	Brain abscess	Trauma	Culture	Paine et al. (23)
2	29	M	Abscess	Brain abscess	Trauma	Culture	Payan et al. (24)
3	76	M	CSF	Meningitis	Neurosurgery	Culture	Mcmahon et al. (25)
4	18	F	CSF	Meningitis	Trauma	Culture	Cohen et al. (26)
5	22	F	Abscess	Brain abscess	Post-partum	ELISA	Zheng et al. (27)
6	40	F	Abscess	Brain abscess	Neurosurgery	16S PCR, Culture	House et al. (28)
7	17	F	CSF, subdural hematoma	Infected subdural hematoma, bacteremia	Post-partum	Culture, Blood culture	Douglas et al. (29)
8	40	M	Brain abscess	Brain abscess	Trauma	16S PCR	Kupila et al. (30)
9	48	M	Brain abscess	Brain abscess	Neurosurgery	16S PCR	Mccarthy et al. (31)
10	17	F	Brain abscess	Brain abscess	Trauma	Culture	Mccarthy et al. (31)
11	41	F	Brain abscess	Brain abscess	Uterine curettage	16S PCR	Al Masalma et al. (32)
12	48	F	CSF	Meningitis	Neurosurgery	16S PCR	Lee et al. (33)
13	40	M	Brain abscess	Brain abscess	Trauma	16S PCR	Henao-Martinez et al. (34)
14	26	M	Blood hip joint and CSF	Disseminated *M. hominis* infection	Immunodeficiency	Culture	Sato et al. (35)
15	17	M	Spinal abscess	Spinal abscess	Trauma	Culture	Whitson et al. (2)
16	43	M	Intracranial sensor	Subdural hematoma	Trauma	MALDI-TOF MS	Pailhories et al. (20)
17	21	F	Spinal abscess	Spinal abscess post epidural anesthesia	Post-partum	Culture 16S PCR	Hos et al. (36)
18	39	M	CSF	Meningitis	Trauma	Quantitative PCR Culture MALDI-TOF MS	Reissier et al. (21)
19	71	M	CSF	Meningitis	Neurosurgery	16S PCR	Zhou et al. (37)
20	57	M	Brain abscess	Brain abscess	Neurosurgery	MALDI-TOF MS	Bregin et al. (38)
21	25	F	Empyema	subdural empyema and cerebritis	Trauma	Culture 16S PCR	Qamar et al. (39)

*CSF, cerebrospinal fluid; ELISA, Enzyme-Linked Immunosorbent Assay; MALDI-TOF MS, matrix-assisted Laser desorption/ionization time-of-flight mass spectrometry; PCR, polymerase chain reaction*.

**Table 2:** Pediatric cases of *M. hominis* CNS infection.

**Case**	**Sex**	**Age**	**Sample site**	**Diagnosis**	**Gynecological status**	**Diagnosis method**	**References**
1	F	5 days	CSF	Meningitis	PROM	Culture	Gewitz et al. (40)
2	F	9 days	CSF	Meningitis	Normal birth	Culture	Gewitz et al. (40)
3	F	4 h	CSF	Meningitis, GBS sepsis	PROM	Culture	McNaughton et al. (41)
4	M	12 days	CSF	Meningitis	Preterm (30 weeks)	Culture	Grist et al. (42)
5	M	3 weeks	Brain abscess	Brain abscess	Normal	16S PCR	Rao et al. (43)
6	M	Birth	CSF	Meningitis	Maternal fever during birth	16S PCR	Wolthers et al. (44)
7	F	25 days	CSF	Meningitis	Normal	16S PCR	Hata et al. (45)
8	M	Birth	CSF	Meningitis	PPROM Preterm (24 weeks)	Culture	Watson et al. (46)
9	M	Birth	CSF	Meningitis	Preterm (26 weeks)	16S PCR	Watt et al. (47)
10	F	Birth	Blood + CSF	Sepsis, meningitis	Not specified	Culture	Huang and Mu (48)
11	F	11 days	CSF	Meningitis	Normal	16S PCR	Wildenbeest et al. (49)
12	F	6 years	CSF	VP shunt infection	Non-Relevant	16S PCR	Sato et al. (50)
13	M	87 days	CSF	Meningitis (post VP shunt insertion)	PPROM Pre-term (33 weeks)	16S PCR	Taku et al. (51)

*CSF, cerebrospinal fluid; PROM, premature rupture of the membranes; PPROM, preterm premature rupture of the membranes; GBS, group-B streptococcus; PCR polymerase chain reaction, VP ventriculo-peritoneal*.

In adults, *M. hominis* was identified in the CSF in eight of the cases (38.1%). Twelve of the cases described had brain abscess with *M. hominis* (57.1%), one had subdural empyema similar to our patient's (4.8%), and two cases described spinal abscesses (9.5%). We found a single case (35) of an adult immunodeficient patient with hypogammaglobulinemia that presented with disseminated *M. hominis* infection in blood, CSF, and synovial fluid (4.8%). Microbiological diagnosis of *M. hominis* infection was made by culture in 12 cases (57.1%), by 16S PCR in eight cases (38.1%) and by MALDI-TOF-supplemented culture in three cases (14.2%). Twelve of the thirteen pediatric cases described (Table 2) were in neonates (92.3%), in which translocation from the mother's birth canal may explain the occurrence of *M. hominis* in the CSF. A single case of non-neonate was described (50)—in which no predisposing factor was identified. Microbiological diagnosis was made by culture in six of the cases (46.1%), and by 16S PCR in the other seven cases (53.8%).

## Discussion

This case depicts an immunocompetent young patient suffering CNS infection and multisite surgical infection with *M. hominis* following trauma. The diagnosis was facilitated by MALDI-TOF MS, thereby enabling relatively rapid and specific treatment that was accompanied by major neurological improvement. Upon reviewing past cases, some important issues arise regarding CNS infections with *M. hominis*: risk factors and their connection to the pathogenesis, diagnostic tools, and rapidity of diagnosis. These issues potentially affect the clinical outcomes of these patients.

Adults presenting with secondary CNS infection can be divided to three groups according to pre-disposing factors: post-partum female patients, multi-trauma patients, and patients undergoing neurosurgical interventions. *M. hominis* asymptomatically inhabits the GU tract in about 15% of adults (38), and more rarely inhabits the respiratory mucosa of healthy adults. CNS infection caused by *M. hominis* is thought to be caused by either direct contamination during trauma or surgery or by secondary dissemination from genitourinary manipulation or disruption of colonized airway by intubation. It seems that in all these patients, the common denominator is the genitourinary tract manipulation. This manipulation, either during delivery or by urethral catheterization might cause transient migration of *M. hominis* into the blood stream, which may, in rare cases, result in extragenital infection. In the setting of head-trauma or neurosurgical interventions, urinary catheterization is often performed urgently and with sub-optimal attention to aseptic conditions, which may increase the risk for metastatic infection.

In the literature review we performed, only in a single case *M. hominis* was isolated from the bloodstream, meaning that the translocation occurring after GU manipulation may lead to an asymptomatic phase of bacteremia (28), with clinical manifestations only occur once the bacteria inhabit the specific infected tissue such as joints, CNS, surgical site etc.

This hypothesized mechanism, however, does not explain of course the paucity of CNS infections caused by *M. hominis*, and even less the fact that other organisms that commonly inhabit the genitourinary tract, are not implicated in CNS infections in the same risk groups. Interestingly, a recent case-report by Xing et al. described a case of post-surgical *Ureaplasma parvum* meningitis, which was diagnosed by metagenomic next-generation sequencing (52). This genitourinary bacteria was not described in association with CNS infections in adults so far, and the clinical significance of this diagnostic method is unclear yet. Considering this, other factors, not yet known, seem to have an important role in the pathogenesis of *M. hominis* in CNS infections, and further research is needed to unveil them.

The nature of this cell-wall free bacterium causes difficulties in timely diagnosis. The organism is not revealed by the Gram stain, and although it does grow on routine media, growth is slow, resulting in significant delays. Additionally, its rarity in CNS infection has not encouraged the inclusion of the organism in rapid molecular diagnostic routines such as multiplex PCR. MALDI TOF identification has mitigated the delay in diagnosis to some extent. Its use was described in three earlier cases of CNS infection caused by *M. hominis*, with a faster time-to diagnosis in these cases compared to the other cases. In the cases reviewed here in adults, the mean time to microbiological diagnosis was 22 days. Due to the fact that *M. hominis* can grow on blood agar, manifesting usually as small pinpoint colonies, in the setting of a patient with suspected CNS infection and negative CSF gram stain, especially among the three groups discussed, a high level of suspicion should arise, and agars should be incubated for 72–96 h (53) at least and inspected closely in order not to overlook these colonies and discard the culture as negative.

Infection of the CNS with *M. hominis* usually presents as meningitis, which is commonly treated empirically with antibiotics such as β-lactams and glycopeptides that act on the bacterial cell wall. *M. hominis* is thus intrinsically resistant to these antibiotics, rendering such empirical treatment ineffective. Appropriate microbial agents for *M. hominis* includes tetracyclines, fluoroquinolones, or chloramphenicol, which are usually not prescribed prior to a confirmed microbiological diagnosis. These two facts contribute to the poor prognosis attributed to patients with CNS infection with *M*. *hominis*. In the cases reviewed, three resulted in death (15%), and four others in severe morbidity (20%).

There are major points that might improve the outcomes of *M. hominis* central nervous system infections. First, a high index of suspicion is required in patients with the predisposing factors, such as neurosurgical intervention, trauma or post-partum, especially if CSF findings display polymorphonuclear pleocytosis with a negative gram stain with late-appearing pinpoint colonies on blood agar and no alternative diagnosis. This situation warrants an alert to the laboratory for prolonged incubation and close inspection of culture media. In such cases, empirical addition of a suitable tetracycline to the treatment should be considered early in the course of the disease. Our experience with MALDI TOF is consistent with the observation that it substantially reduces the time to microbiological diagnosis of *M*. *hominis* infections, thereby improving clinical outcomes. Second, alerting the laboratory might trigger the deployment of more rapid and accurate microbiological methods, thus avoiding the inherent delays of conventional diagnosis. In conclusion, this rare case of *M. hominis* subdural empyema following decompressive craniotomy highlights the necessity for fusing conventional routines and advanced laboratory capabilities to mitigate delays due to the former and promote targeted early application of the latter. We suggest that this combined approach will optimize earlier diagnosis and effective therapeutic intervention in unusual, complicated infections.

## Data Availability Statement

The original contributions presented in the study are included in the article/supplementary material, further inquiries can be directed to the corresponding authors.

## Ethics Statement

The studies involving human participants were reviewed and approved by the Hadassah Medical Center Ethics Committee (HMO-460-12), including a waiver of informed consent. Written informed consent for participation was not required for this study in accordance with the national legislation and the institutional requirements.

## Author Contributions

RN-P, YO, and AP: initiation, literature review, and drafting. YO, OA, and GR: revisions. OA, AM-G, VT, and MA: lab data. All authors contributed to the article and approved the submitted version.

## Conflict of Interest

The authors declare that the research was conducted in the absence of any commercial or financial relationships that could be construed as a potential conflict of interest.

## Publisher's Note

All claims expressed in this article are solely those of the authors and do not necessarily represent those of their affiliated organizations, or those of the publisher, the editors and the reviewers. Any product that may be evaluated in this article, or claim that may be made by its manufacturer, is not guaranteed or endorsed by the publisher.
